# Transient comparison of techniques to counter multi-drug resistant bacteria: prime modules in curation of bacterial infections

**DOI:** 10.3389/frabi.2023.1309107

**Published:** 2024-01-26

**Authors:** Muhammad Naveed, Muhammad Waseem, Izma Mahkdoom, Nouman Ali, Farrukh Asif, Jawad ul Hassan, Hamza Jamil

**Affiliations:** ^1^Department of Biotechnology, Faculty of Science and Technology, University of Central Punjab, Lahore, Pakistan; ^2^National Institute of Biotechnology and Genetic Engineering, Faisalabad, Punjab, Pakistan

**Keywords:** multi-drug resistant bacteria, staphylococcus aureus, vancomycin-resistant enterococcus, medicinal plants, bacterial vaccines

## Abstract

Multidrug-resistant organisms are bacteria that are no longer controlled or killed by specific drugs. One of two methods causes bacteria multidrug resistance (MDR); first, these bacteria may disguise multiple cell genes coding for drug resistance to a single treatment on resistance (R) plasmids. Second, increased expression of genes coding for multidrug efflux pumps, which extrude many drugs, can cause MDR. Antibiotic resistance is a big issue since some bacteria may withstand almost all antibiotics. These bacteria can cause serious sickness, making them a public health threat. Methicillin-resistant *Staphylococcus aureus* (MRSA), vancomycin-resistant *Enterococcus* (VRE), Multidrug resistant *Mycobacterium tuberculosis* (TB), and CRE are gut bacteria that resist antibiotics. Antimicrobial resistance is rising worldwide, increasing clinical and community morbidity and mortality. Superbugs have made antibiotic resistance in some environmental niches even harder to control. This study introduces new medicinal plants, gene-editing methods, nanomaterials, and bacterial vaccines that will fight MDR bacteria in the future.

## 1 Introduction

Antimicrobial resistance poses a critical global health challenge, driven by the emergence of multidrug-resistant organisms, particularly bacteria. These organisms have developed the ability to withstand the effects of antibiotics that were once effective in controlling and killing them. The widespread use of antibiotics in human therapy, agriculture, and aquaculture has contributed to the selection of pathogenic bacteria that are now resistant to multiple drugs (Varela et al., 2021). Bacteria acquire multidrug resistance through two primary mechanisms. First, they can accumulate multiple genes, each conferring resistance to a single drug, within a single cell. This accumulation often takes place on resistance (R) plasmids. Second, multidrug resistance can also occur due to increased expression of genes encoding multidrug efflux pumps, enabling the extrusion of a broad range of drugs (Bush and Bradford, 2019). For instance, Staphylococcus aureus, commonly known as MRSA (*Methicillin-Resistant Staphylococcus aureus*), and *Neisseria gonorrhoeae*, the causative agent of gonorrhea are now almost universally resistant to benzylpenicillin, a drug previously effective in controlling these infections (Ahmadi et al., 2022). These bacteria can cause severe diseases and are resistance to nearly all available antibiotics, presenting a significant public health challenge. Examples of these superbugs include methicillin-resistant *Staphylococcus aureus* (MRSA), vancomycin-resistant *Enterococcus* (VRE), multidrug-resistant *Mycobacterium tuberculosis* (TB), and carbapenem-resistant Enterobacteriaceae (CRE) gut bacteria (Mazumdar and Adam, 2021).

The global burden of antimicrobial resistance has led to increased morbidity and mortality in clinical and community settings. The spread of antibiotic resistance to various environmental niches and the emergence of superbugs have complicated effective control strategies. In response to this crisis, international, national, and local approaches have been recommended to control and prevent antimicrobial resistance (El-Halfawy et al., 2020). Key strategies to combat antimicrobial resistance include the rational use of antimicrobials, the regulation of over-the-counter availability of antibiotics, improved hand hygiene, and infection prevention. Addressing this challenge requires a multidisciplinary, collaborative regulatory approach (Liu et al., 2021).

Antimicrobial resistance is now recognized as a global issue by various stakeholders. In 2011, the World Health Organization (WHO) designated it as a major theme, drawing international attention to the need for collective efforts in addressing AMR (Ahmed and Baptiste, 2018). WHO has put forth recommendations that emphasize increased collaboration among governments, non-governmental organizations, professional groups, and international agencies, the establishment of networks for AMR surveillance, international efforts to combat counterfeit antimicrobials, incentives for the development of new drugs and vaccines, and the strengthening of existing programs to contain AMR (Fiore et al., 2019).

This study specifically explores the pathogenic mechanisms of some of the most lethal multidrug-resistant bacteria, including Vancomycin-Resistant *Enterococci* (VRE), Methicillin-Resistant *Staphylococcus aureus* (MRSA), Extended-Spectrum β-Lactamase (ESBLs) producing Gram-negative bacteria, *Klebsiella pneumoniae carbapenemase* (KPC) producing Gram-negative bacteria, and Multidrug-Resistant Gram-Negative Rods (GNR). Researchers are harnessing advancements in science and technology to combat these bacteria using innovative approaches such as newly discovered medicinal plants, gene-editing techniques, novel nanomaterials, and bacterial vaccines. This review provides insights into the mode of action of these techniques in neutralizing bacterial infections, offering valuable guidance for researchers seeking effective strategies to combat multidrug-resistant bacteria in the future. By presenting these critical aspects of antimicrobial resistance in a structured manner, this information aims to enhance reader comprehension and awareness of the global challenge posed by multidrug-resistant organisms.

## 2 Multi-drug resistant bacteria

### 2.1 Vancomycin-resistant *Enterococci*

*Enterococci* are facultative anaerobes, ovoid-shaped gram-positive bacteria, and commensal inhabitants of the gastrointestinal tract of humans (Fiore et al., 2019). Impulsive applications of antibiotics have prompted the adaptive resistance mechanism in *Enterococci* and modulated it into an emerging nosocomial pathogen along with urinary tract and bloodstream infections (Said and Abdelmegeed, 2019). The discovery of antibiotics was intended to guarantee defense against pathogenic bacteria, but after some optimistic period, bacteria, for instance, *Enterococci*, acquired resistance against penicillin and aminoglycosides modified penicillin, streptomycin. Metabolic modifications, hypermutability, and mobile genetic factors in *Enterococci* acquired genes are involved in the Vancomycin resistance mechanism (Sun et al., 2018). *Enterococci* have become an opportunistic pathogen with intrinsic and acquired resistance against quinupristin, dalfopristin, tigecycline, and vancomycin antibiotics (Ahmed and Baptiste, 2018).

#### 2.1.1 Pathogenesis of VRE

**Figure 1:**
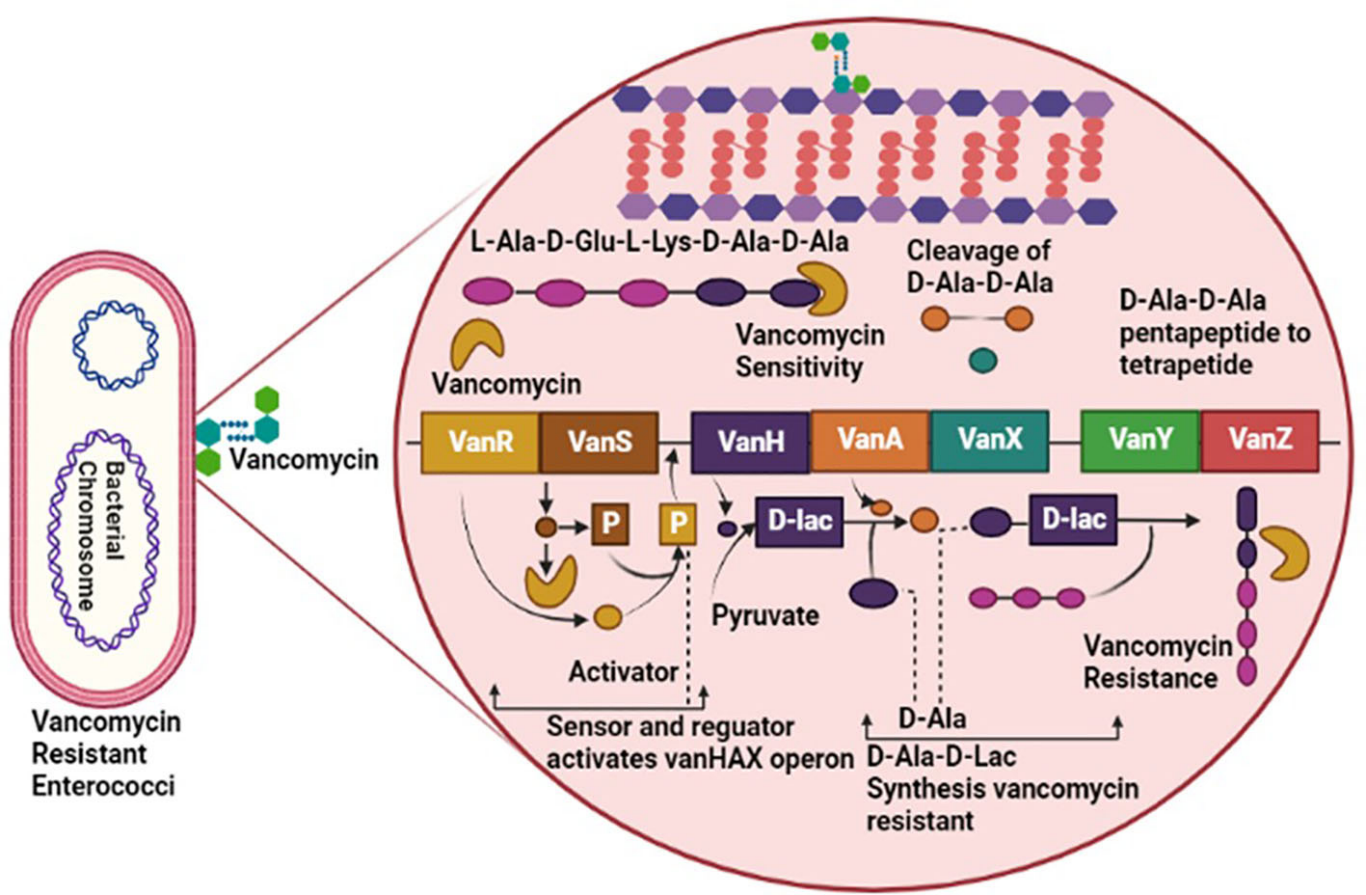
Pathogenesis of Vancomycin-Resistant *Enterococci* (VRE), involving *VanR*, *VanS, VanH, VanA, VanX, VanY* and *VanZ* virulent factors causing resistance against vancomycin antibiotic.

*Enterococci* have a diverse resistance mechanism against antibiotic agents, including resistance against antibiotics interfering with cell wall components, protein synthesis, nucleic acid replication, and synthesis (Sianglum et al., 2019). Generally, the Cell wall of *Enterococci* is synthesized by crosslinking of peptidoglycan’s D-alanine-D-alanine agents, a terminal of pentapeptide precursor. Vancomycin performed its inhibitory activity by binding with the D-alanine-D-alanine of the cell wall and obstructing cell wall synthesis (Turner et al., 2021). However, vancomycin-resistant *Enterococci* have adapted to bind the vancomycin with D-lactate by altering the pentapeptide precursors of the cell wall. Modification in precursors has reduced the binding affinity of vancomycin to D-alanine by about 1000-fold. The entire mechanism of vancomycin resistance requires seven enzymes: VanH, VanX, VanY, VanZ, VanR, and VanS, taking VanA as a model for altering and eradicating a normal precursor, D-alanine (**Figure 1**) (Mühlberg et al., 2020).

### 2.2 Methicillin-resistant *Staphylococcus aureus*

*Staphylococcus aureus* is an emerging, facultative gram-positive opportunistic bacteria associated with various community-acquired and nosocomial infections (Waseem et al., 2023). It has also been reported with serious skin infections, pneumonia, and toxic shock syndrome (Faron et al., 2016). *Staphylococcus aureus* is a versatile pathogen with broad-spectrum hosts from sheep, pigs, and cattle to poultry. Intrinsic and adaptive characteristics of *S. aureus* have produced resistance and tolerance mechanisms against a range of antibiotics from narrow to broad-spectrum, including Penicillin, Quinolone, Methicillin, and Vancomycin (Gajdács, 2019). This bacterium encodes hydrolyzing enzymes to generate an acquired resistance mechanism against antibiotics and is illustrated as multidrug resistance bacteria (MDR) (Chojnacki et al., 2019).

#### 2.2.1 Pathogenesis of MRSA

Methicillin-resistant *Staphylococcus aureus* (MRSA), a multi-drug resistant evolving pathogen, has acquired resistance against frequently modified antibiotics. MRSA performed its activity by modifying penicillin-binding proteins (PBP2) (El-Halfawy et al., 2020). Normal PBP2 and bifunctional transglycosylase-transpeptidase are involved in peptidoglycan biosynthesis (cell wall). Here, MRSA produces modified genes that encode PBP2a responsible for the inactivation of transpeptidase (TP) of normal PBP2 and turn off the reach of β-lactams to the serine active site of TP, leading to the non-susceptibility of β-lactams (Dawan and Ahn, 2020).

**Figure 2:**
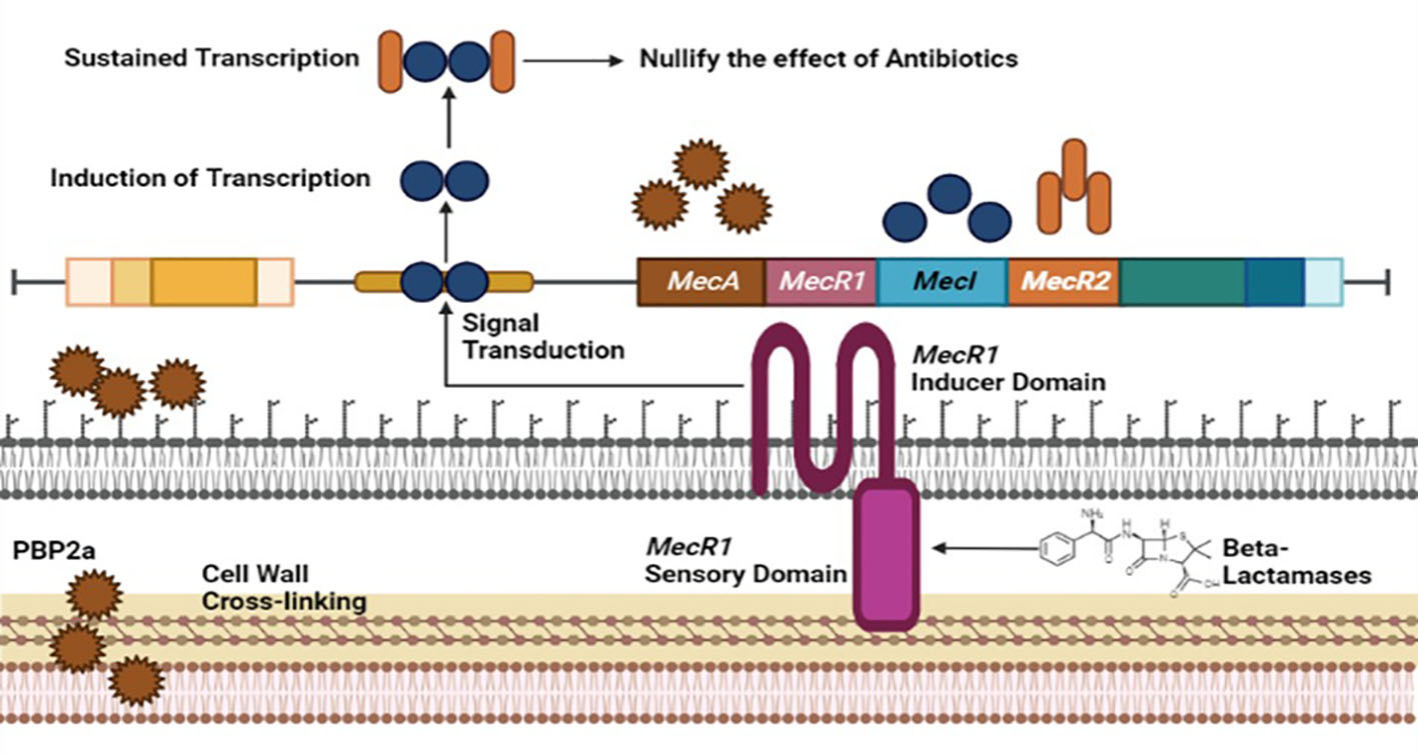
Resistance mechanism of *Staphylococcus aureus* against Methicilin antibiotic involving *MecA, MecR1, MecI* and *MecR2* genome factors of MRSA.

The Gene involved in the expression of PBP2a is *mecA*, found in *staphylococcal* chromosome cassette, regulated by MecIR proteins. These proteins MecIR regulate the expression of the mecA gene for the production of PBP2a on exposure to β-lactams. B-lactams have inherited abilities to counter *mecA* expression by activating β-lactamase regulators *BlaI* and *BlaR* (Mazumdar and Adam, 2021). The resistance mechanism of MRSA could be heterogeneous. Most cells express low resistance and could be homogenous, in which most cells show high resistance but are in the minority. Chromosomal alterations can improve the regulation of the mecA gene to produce additional PBP2a for a high homogenous resistance mechanism in MRSA. Introducing new β-lactams, including fifth-generation cephalosporins and ceftobiprole, may inhibit the PBP2a of methicillin-resistant *staphylococcus aureus* (**Figure 2**) (El-Halfawy et al., 2020).

### 2.3 Extended-spectrum β-lactamase producing Gram-negative bacteria

Multi-drug resistance in bacteria, facilitated by factors like uncooked food, contact with animals, and antibiotic misuse, poses a global threat to combating microbial infections (Fisher and Mobashery, 2020). Antibiotics are potentially significant in preventing the spread of communicable diseases globally (Khan et al., 2020). Gram-negative bacteria like *Escherichia coli*, *Pseudomonas aeruginosa*, *Klebsiella pneumoniae*, and *Acinetobacter* spp. are responsible for various infections. They produce β-lactamase enzymes, a major cause of resistance, which can hydrolyze β-lactam antibiotics (Hernando-Amado et al., 2019; Liu et al., 2019). This is a serious threat to underdeveloped countries with less life-saving and diagnostic surveillance having a high burden of microbial infections. Gram-negative bacteria such as *Escherichia coli*, *Pseudomonas aeruginosa*, *Klebsiella pneumoniae*, and *Acinetobacter* spp. are pathogenic microbes which are responsible for causing abdominal, blood and urinary tract infections (Monserrat-Martinez et al., 2019).

These gram-negative bacteria contain a β-lactamase enzyme that hydrolyzes an integral part of the ring of β-lactam antibiotics. It is a major factor that develops beta-lactam resistance (Portsmouth et al., 2018). Extended spectrum β-lactamases (ESBLs) are a diverse group of enzymes that can hydrolyze penicillin and cephalosporins but not carbapenems and cephamycins. This resistance is a significant concern, especially in underdeveloped countries with limited healthcare resources (Davies and Everett, 2021). These Extended spectrum β-lactamases are inhibited by tazobactam, sulbactam and clavulanic acid (Bush and Bradford, 2019). The most common among the four classes of β-lactamase (A, B, C and D) are CTX-M, TEM, and SHV families (Ali et al., 2018).

#### 2.3.1 Pathogenesis of ESBLs

ESBL production is prevalent in *Klebsiella* pneumonia and *Escherichia coli* strains, with over 400 known types of β-lactamases. Class A β-lactamases, like those in the TEM and SHV families, can hydrolyze various antibiotics but are inhibited by clavulanic acid, carbapenems, and cephamycins (Bush, 2018; Hammoudi Halat and Ayoub Moubareck, 2020; Yang et al., 2020; Ejaz et al., 2021). However, these enzymes can be inhibited by using clavulanic acid or by treating the microorganism with carbapenems (imipenem) and cephamycins (cefoxitin) (Ramos et al., 2020). CTX-M β-lactamases are efficient against certain antibiotics and have various subtypes CTX-M, CTX-M-2, CTX-M-3 and CTX-M-14 (Carcione et al., 2021; Castanheira et al., 2021).

Chromosomes of some bacteria and various *Enterobacteriaceae* encode for class C AmpC β-lactamases. They favor the bacteria by developing resistance against most penicillins, cephalothin, cefoxitin, cefazolin, and different β-lactamase inhibitors (Adel et al., 2021; Farhat et al., 2022). Overexpression of AmpC enzymes leads to cephalosporin resistance in a broad spectrum, for example, ceftriaxone, ceftazidime, and cefotaxime. AmpC-producing bacteria can be treated effectively using Carbapenems, but in some studies, resistance against carbapenem in the microbes has also been reported (Meini et al., 2019; Ali et al., 2020).

**Figure 3:**
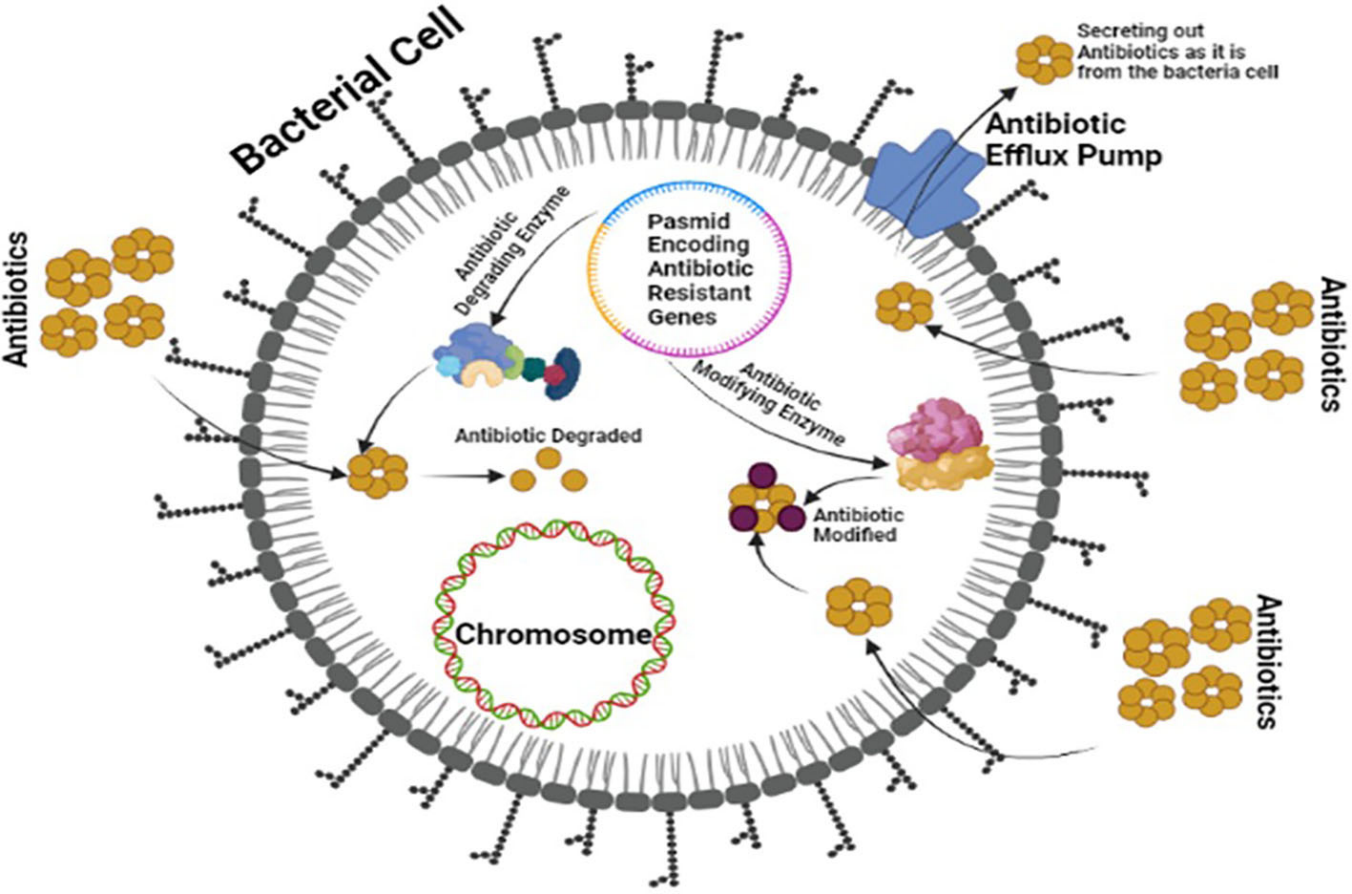
Multi-Drug Resistant Mechanism of ESBLs showing a plasmid encoding antibiotic resistance and modifying enzymes by bacteria against board spectrum antibiotics.

OXA-type class D β-lactamases can hydrolyze carbapenems and are plasmid-encoded. Some bacteria, including Pseudomonas aeruginosa, produce uncommon ESBLs like GES, PER, VEB, and IBC β-lactamases (Agyepong, 2017; Govindaswamy et al., 2019). The plasmid of bacteria, mainly *P. aeruginosa*, also produces uncommon ESBLs such as GES, PER, VEB, and IBC β-lactamases (Sawa et al., 2020). Class B MBLs can become carbapenemases and are transferable among bacteria (Khoshbakht et al., 2014). **Figure 3** illustrates the production of ESBLs and the inhibition of β-lactam ring-containing antibiotics by gram-negative bacteria.

### 2.4 Klebsiella pneumoniae carbapenemase producing Gram-negatives

Gram-negative bacteria are rapidly becoming resistant to carbapenem worldwide. Specifically, these carbapenemase-producing bacteria mainly originate from healthcare centers. Klebsiella pneumoniae carbapenemase (KPC)-producing Klebsiella pneumonia (KPC-KP) is a widely reported strain causing carbapenem resistance (Berglund et al., 2017). As a result of the rapid increase in resistance against cephalosporin antibiotics within Enterobacteriaceae, some drugs have become commonly to be used worldwide, such as biapenem, meropenem, ertapenem, imipenem, doripenem and panipenem. Carbapenem antibiotics initiate bactericidal activity by binding irreversibly with an amino acid Ser403 present within the active site of the penicillin-binding protein of bacteria (Hussain et al., 2021). This inhibits the bacterial cell wall synthesis, resulting in the bacterial cell being killed. The blaKPC gene responsible for carbapenem resistance is present on mobile genetic elements, facilitating the widespread of carbapenem resistance among intra and interspecies (Mora-Ochomogo and Lohans, 2021). Similarly, the extended-spectrum β-lactamases (ESBLs) and KPCs are efficient enough to hydrolyze β-lactam rings in monobactams, cephalosporins, and penicillins. *In vitro* studies suggest that KPC enzymes have the highest affinity for ertapenem antimicrobial (Caprari, 2021).

#### 2.4.1 Pathogenesis of antibiotic resistance in carbapenem-resistant *Enterobacteriaceae*

**Figure 4:**
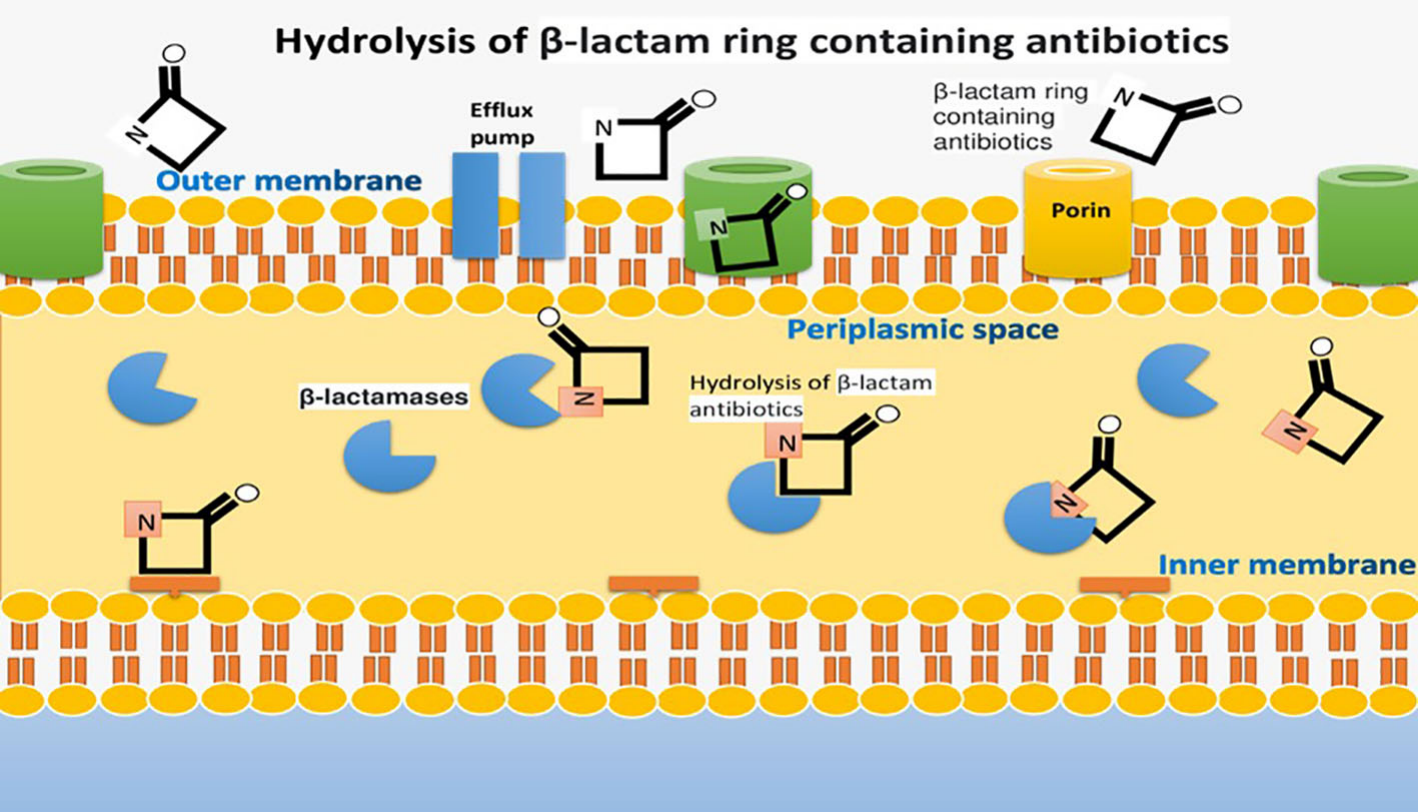
Pathogenesis of Carbapenem-Resistant *Enterobacteriaceae* causing multi-drug resistance against Carbapenem Antibiotics.

Carbapenem resistance in *Enterobacteriaceae* is primarily caused by three mechanisms: carbapenem degradation using the enzyme Carbapenemase, activation of efflux pumps, and alterations in porin proteins (Bianco et al., 2021). Carbapenemase production, along with β-lactamases and porin mutations, leads to resistance. KPC enzymes play a significant role in this resistance, with different subtypes available (Pulzova et al., 2017; Chauzy et al., 2019; Brindangnanam et al., 2022). Bacteria can also use other mechanisms, like efflux pump activation and changes in drug-binding proteins, to resist carbapenems. Plasmid-encoded carbapenemases facilitate resistance spread among bacteria (Seifert et al., 2018; Palacios et al., 2020). In Pseudomonas aeruginosa, efflux pump overexpression and porin loss contribute to carbapenem resistance. Gram-negative bacteria are generally more resistant due to their cell wall, reduced permeability, efflux pumps, and broad-spectrum β-lactamases (Galani et al., 2021; Zhang et al., 2022). Some traditional treatments may be ineffective, highlighting the need for new antimicrobials to combat these resistant infections (Thaden et al., 2017). The pathogenesis of carbapenem-resistant Enterobacteriaceae, which results in multi-drug resistance against carbapenem antibiotics, is depicted in **Figure 4**.

### 2.5 Multidrug-resistant Gram-negative rods

Gram-negative rod-shaped bacteria, including *Escherichia coli, Acinetobacter baumannii, Pseudomonas aeruginosa*, and *Enterobacteriaceae*, are inhabitants of human skin and the GI tracts of humans, animals, sewage, and soil. These are particularly involved in urinary tract infections, community-acquired and nosocomial infections (Zrelovs et al., 2021). Gram-negative bacteria have developed numerous resistance mechanisms against multiple antibiotics owing to genetic variations and inherent and acquired characteristics. Contrary to Gram-positive bacteria, Gram-negative bacteria have a thick outer membrane having porins protecting them from antibiotics by producing antibiotic resistance. Gram-negative bacteria resist several antibiotics, including Carbapenems, piperacillin, ciprofloxacin, broad-spectrum penicillins, and aminoglycosides (Wolfensberger et al., 2019).

#### 2.5.1 Pathogenesis of Multidrug resistant GNR

Gram-negative bacteria exhibit a range of resistance mechanisms; for instance, *E. coli* is a commensal bacterium of the GI tract that produces virulence factors causing sepsis, abdominal infections, and bacteremia (Kabanangi et al., 2021). MDR *E.coli* has adapted to extended-spectrum β-lactam producing β-lactamase (ESBL), hydrolyzing the β-lactam antibiotics. Further, MDR *E.coli* ST131 producing ESBL causing urinary tract infections shows resistance to third-generation cephalosporins and quinolones. Similar to Gram-negative *Pseudomonas aeruginosa* and *Enterobacteriaceae*, ESBL and Carbapenemase-producing Enterobacteriaceae (CPE) production by these gram-negative bacteria resist the antibiotic activities of carbapenems, aztreonam, and cephalosporin.

**Figure 5:**
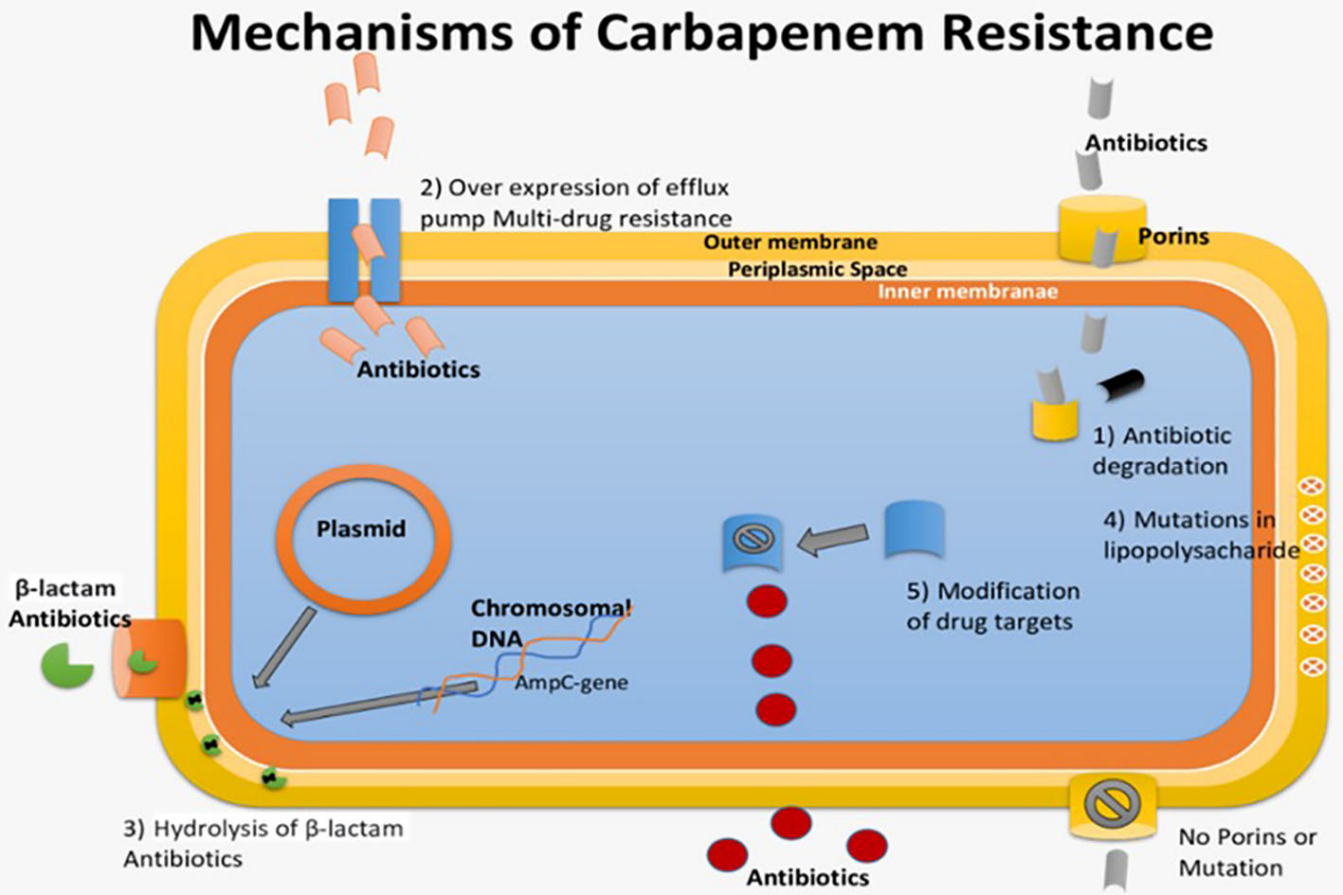
Pathogenesis of Multi-drug resistant GNR causing resistance against antibiotics by gram-positive bacterial species.

The general mechanism of extended-spectrum beta-lactamase production is diverse, and different strategies are applied (Eichenberger and Thaden, 2019). For instance, hydrolysis of ester-amides bond in antibiotics like cephalosporins, redox reaction of tetracycline by TetX enzyme activity and transferases enzymes inhibit the antibiotic activity by switching it with the acetyl or phosphoryl groups. The thick outer membrane of *Pseudomonas aeruginosa* and other gram-negative rods protects them from antibiotic activity. Porins in the outer membrane impede antibiotic activity by reducing their numbers or modifications and adaptations, resulting in porins loss and thereby hindering antibiotic activity (**Figure 5**) (Jean et al., 2022).

## 3 Advancements to combat multi-drug-resistant bacteria

Microbes possess the ability to neutralize antimicrobial activity through various molecular mechanisms. The antimicrobial agent loses its efficacy as it is deactivated by specific proteins produced by the bacterium. For example, β-lactam antibiotics are hydrolyzed by β-lactamases, with enterobacter spp. producing extended-spectrum β-lactamases (ESBL) that share the same inactivating capability, making them challenging to eliminate (Davies and Everett). Additionally, various enzymes, such as acetyltransferase, phosphotransferase, and adenyltransferase, can deactivate specific antibiotics. In the case of resistance to erythromycin, methylation of an adenine residue in the peptidyltransferase of rRNA 23S reduces its affinity for the antibiotic without affecting protein synthesis. Another notable example is the modification of penicillin-binding proteins (PBPs) by MRSA (Anstey et al., 2019).

To combat these microbial strategies, there is a pressing need for advancements in modern techniques. The development and implementation of cutting-edge technologies are crucial in overcoming microbial resistance. Novel approaches, including gene-editing techniques, innovative nanomaterials, and bacterial vaccines, show promise in the fight against antimicrobial resistance. Moreover, a focus on antibiotic stewardship programs is essential. These programs play a pivotal role in promoting responsible antibiotic use, ensuring judicious prescribing, and mitigating the emergence of resistant strains. Following we discussed some advancements in technology, addressing the ever-evolving challenges posed by microbial resistance to antimicrobial agents.

### 3.1 Fifth generation antibiotics

Fifth-generation cephalosporins are a class of antibiotics that belong to the cephalosporin family. They are part of the beta-lactam group of antibiotics and have a broad spectrum of activity against various types of bacteria (Rusu and Lungu, 2020). Fifth-generation cephalosporins are among the newest cephalosporins, and they have been developed to address antibiotic resistance and target a wide range of both Gram-positive and Gram-negative bacteria. Fifth-generation cephalosporins are generally effective against a variety of pathogens, including those resistant to earlier generations of cephalosporins and some other classes of antibiotics (Lin and Kück, 2022). They are valuable additions to the armamentarium of antibiotics available to healthcare providers and are particularly useful in the treatment of complicated infections caused by a range of bacteria (Fong, 2023).

#### 3.1.1 Mode of action of antibiotics

**Figure 6:**
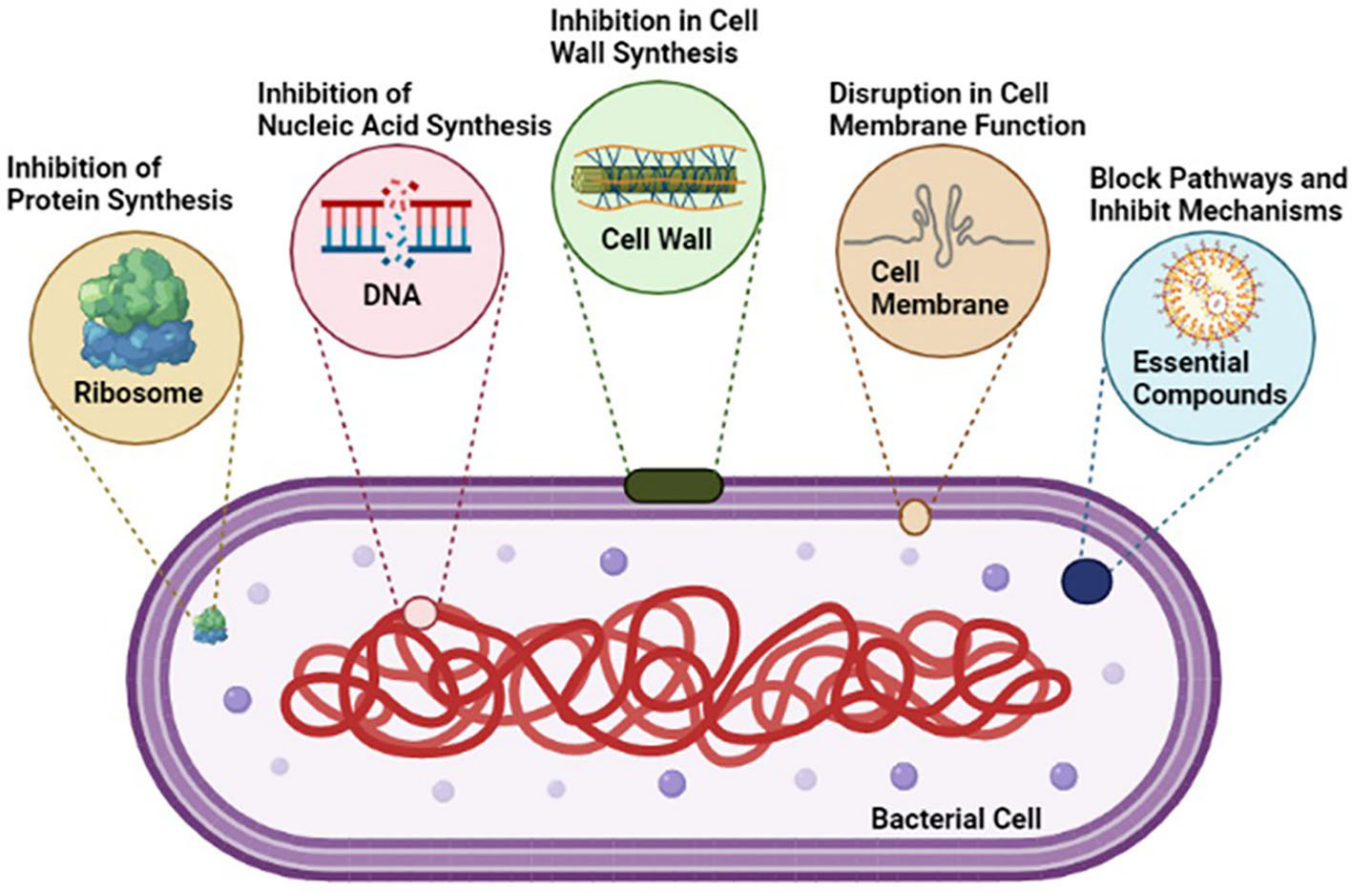
Various Destructive mechanisms of antibiotics against pathogenic bacteria.

Modifications in the host cell surface pose a challenge to the effective penetration of antimicrobials. In Gram-negative bacteria, resistance may arise from alterations in porins or proteins, either through modification or quantitative reduction, which are essential for the entry of many antimicrobial drugs (Dowling et al., 2017). These modifications can impede the incoming flow of various antibiotics by imposing restrictions based on factors such as atomic size, hydrophobicity, and charge, thereby contributing to the intrinsic resistance of numerous microorganisms. An illustrative case of *Pseudomonas aeruginosa*, showcasing resistance to imipenem due to such surface modifications in **Figure 6** (Farhat et al., 2020; Shukla et al., 2020).

Ceftaroline, a cephalosporin antibiotic belonging to the fifth generation, has demonstrated exceptional efficacy in inhibiting the activity of PBP2a, or penicillin-binding protein 2a. This protein plays a pivotal role in conferring resistance to beta-lactam antibiotics in methicillin-resistant *Staphylococcus aureus* (MRSA) and related Gram-positive bacteria. Ceftaroline’s effectiveness stems from its unique structural features and its ability to overcome resistance mechanisms associated with PBP2a (Dafne et al., 2019). Notably, ceftaroline’s success lies in its structural similarity to the D-Ala-D-Ala terminus of the peptidoglycan pentapeptide chain, serving as the natural substrate for PBPs. The beta-lactam ring of ceftaroline is crucial for its mechanism of action, enabling the antibiotic to mimic the peptidoglycan structure and facilitating its binding to the active site of PBP2a (Zhanel et al., 2009).

Upon binding, ceftaroline forms a stable acyl-enzyme complex with PBP2a, distinguishing it from other beta-lactam antibiotics. This stability is attributed to ceftaroline’s unique structural elements, including a 7α-methoxy group and a 3’ amino group. The resulting stable complex effectively inhibits PBP2a, preventing the enzyme from participating in the cross-linking of the peptidoglycan cell wall. This disruption in peptidoglycan synthesis weakens the bacterial cell wall, leading to cell lysis and the eventual demise of MRSA and susceptible Gram-positive bacteria. Crucially, ceftaroline’s ability to overcome PBP2a-mediated resistance makes it a valuable treatment option for infections caused by MRSA and other resistant Gram-positive bacteria (Morosini et al., 2019). In the ongoing battle against antibiotic-resistant microbes, the development and utilization of fifth-generation antibiotics offer a promising avenue for effective treatment. Continued research and innovation in this field are essential to stay ahead of evolving resistance mechanisms and ensure the availability of potent tools to combat challenging microbial infections.

### 3.2 Medicinal plants

From ancient times to the modern era, a plethora of plants, herbs, and shrubs have been harnessed for their therapeutic properties in treating diseases and infections caused by pathogens. These botanical wonders exhibit medicinal and antimicrobial attributes, containing phytochemicals endowed with antioxidant, anti-inflammatory, and antibiotic activities. In the face of escalating bacterial resistance to conventional drugs, a phenomenon known as multidrug resistance, the exploration of alternative sources becomes imperative. Six Cameroonian medicinal plants, namely *Erigeron floribundus*, *Nauclea latifolia*, *Caucalis melanantha*, *Anthocleista schweinfurthii*, *Zehneria scobra*, and *Boehmeria platyphylla* have been investigated for their extracts’ efficacy against multidrug-resistant Gram-positive bacteria. Extracts from these plants exhibited a moderate level of antibiotic activity, with 14.3% of 28 bacterial strains demonstrating minimum inhibitory concentration (MIC) values ranging from 128 to 1024 μg/mL (Djeussi et al., 2016). Beyond antibacterial applications, Cameroonian plants have also demonstrated promise in cancer treatment, exerting cytotoxic effects on cancerous cell lines when phytochemicals from fifteen medicinal plants were applied (Kuete et al., 2016).

In a study, *Artemisia annua* (EtAa, AqAa), *Oxalis corniculata* (EtOc, AqOc), and *A. annua* essential oil (EoAa), along with aqueous and ethanolic extracts, showcased antimicrobial activity. This study focused on multidrug resistance in *Escherichia coli* by determining the minimum bactericidal concentration (MBC) and MIC. Impressively, these extracts inhibited all isolates in 56.7% of cases, presenting a potential alternative to the 13 antibiotics currently employed against EoAa (Golbarg and Mehdipour Moghaddam, 2021). Tuberculosis (TB), a persistent global health concern, has been addressed through the utilization of fifty-two plant species, with Chenopodium ambrosiodes L emerging as the most cited. The historical evolution of TB treatment has witnessed a transition from single-drug therapies to the current approach of multidrug resistance, signifying the adaptation of strategies against the growing drug resistance in the context of tuberculosis (Ahmad et al., 2006; Organization WH, 2013; Nguyen, 2016).

#### 3.2.1 Mode of action of medicinal plants

Medicinal plants harbor phytochemicals with the remarkable ability to suppress and eliminate bacteria through diverse mechanisms. These bioactive compounds target bacterial cells by either breaking down the cell wall or rupturing the cell membrane (Yadav et al., 2023). The process is particularly effective against multidrug-resistant strains, where the interaction with ion channel carrier proteins, specifically efflux pumps, plays a crucial role in acquiring resistance. Upon removal of the cell wall, the bacterial cell becomes vulnerable and succumbs to its demise. Similarly, the rupture of the cell wall exposes the bacterial cell components, leading to the eventual demise of the bacteria (Jorgensen et al., 2017). The intricate mechanism of ion channel efflux, integral to the functionality of resistant drugs, is disrupted by these phytochemicals, rendering them potent agents in combating antibiotic-resistant bacteria (Khameneh et al., 2021). Subsequently, these extracted and refined phytochemicals find application as drugs in medicine, proving effective in suppressing or eradicating bacterial infections (*Escherichia coli*, *Staphylococcus aureus*, and *Mycobacterium tuberculosis*) and offering a promising avenue for addressing antibiotic resistance in a multifaceted manner.

### 3.3 Bacteriocins

Bacteriocins, bacterial ribosomal products, are antimicrobial peptides that serve as a defense mechanism against pathogenic and deteriorating bacteria. These low molecular weight peptides exhibit diverse inhibitory mechanisms against other bacteria, categorizing them into narrow-spectrum bacteriocins, which hinder the growth of bacteria within the same species, and broad-spectrum bacteriocins, which impede bacteria belonging to different species (Negash and Tsehai, 2020). Both Gram-positive and Gram-negative bacteria produce these antimicrobial peptides. The bacterial cells generating bacteriocins possess a specific immune system to resist their own products. Bacteriocins are encoded by a cluster of genes responsible for their immunity, transport, and production. These genes are typically located adjacently on plasmids or chromosomes, expressing in parallel fashion (O’Connor et al., 2020).

The antimicrobial activity of bacteriocins extends to various bacteria, including food-borne pathogens, making them highly applicable in food preservation. An exemplary case is Nisin, an FDA-approved bacteriocin utilized as a food preservative in over 50 countries worldwide, inhibiting the growth of Listeria and numerous other Gram-positive microbes. The potential of bacteriocins to combat antibiotic-resistant bacteria underscores their importance in clinical applications. For instance, griselimycin, a bacteriocin, demonstrates inhibitory effects against *Mycobacterium tuberculosis in vitro* and in mice, offering promise as a treatment for multidrug-resistant strains. Furthermore, bacteriocins exhibit efficacy against diverse viruses, such as dengue viruses and HIV (Yang et al., 2014).

#### 3.3.1 Mode of action of bacteriocin

**Figure 7:**
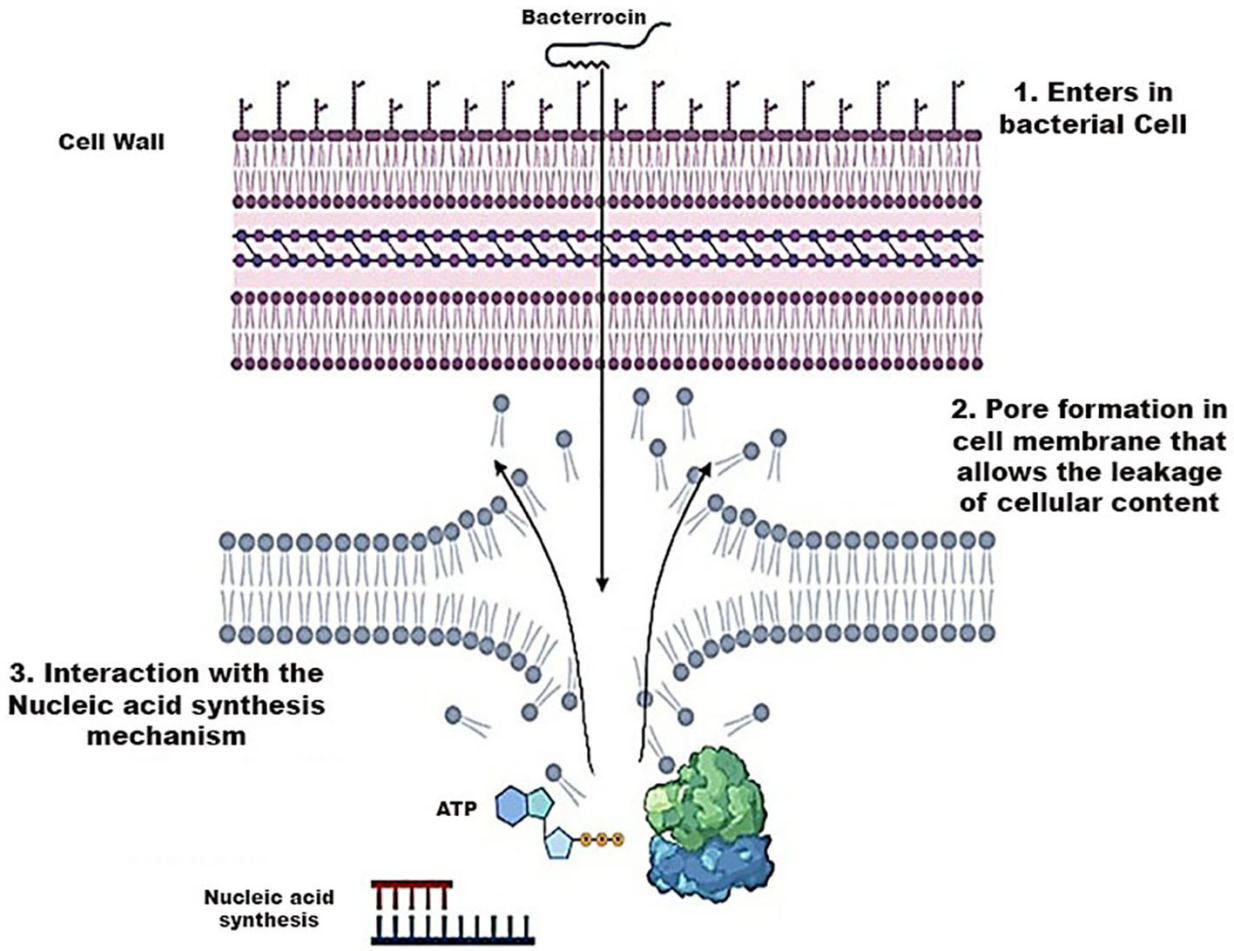
Mechanism of Action of Bacteriocins playing a role in the destruction of bacterial cell wall.

The action of bacteriocins initiates through their interaction with the cell membrane of bacteria. Given the negatively charged nature of the cell membrane, electrostatic interactions occur, leading to both specific and non-specific binding. This interaction facilitates the formation of pores, enabling the insertion of the bacteriocin into the target cell. Subsequently, these pores induce a continuous efflux of cellular components, including ions, amino acids, and ATP, resulting in the leakage of all cellular content and ultimately leading to cell death. Furthermore, this mechanism may induce cell lysis by releasing autolytic enzymes associated with the cell wall lipids of Gram-positive bacteria (van Heel et al., 2017). In certain scenarios, bacteriocins may not exclusively interact with the cell membrane; instead, they can directly interfere with the nucleic acid synthesis mechanism shown in **Figure 7** (Acedo et al., 2015).

### 3.4 Bacterial vaccines

Bacterial vaccines, comprised of antigens either synthetically produced or derived from pathogens, play a crucial role in eliciting an immune response within the body. Upon entry, these vaccines stimulate the immune system to generate antibodies specific to the targeted pathogen, thereby preventing its growth upon future exposure. Notably, bacterial vaccines exhibit an extended lifespan, spanning decades, and exert a profound impact on human health (Poolman, 2020). The probability of resistance emergence against these vaccines within the genome is remarkably low. This phenomenon can be attributed to the concise timing of vaccine administration relative to the pathogen’s lifecycle and the diversity and variability of target sites. The latter necessitates a high rate of mutations for the pathogen to acquire resistance against the vaccine. Bacterial vaccines contribute significantly to reducing mortality and illness associated with bacterial diseases, standing as one of the most efficient medical interventions (Bekeredjian-Ding, 2020).

#### 3.4.1 Mode of Action of bacterial vaccines

**Figure 8:**
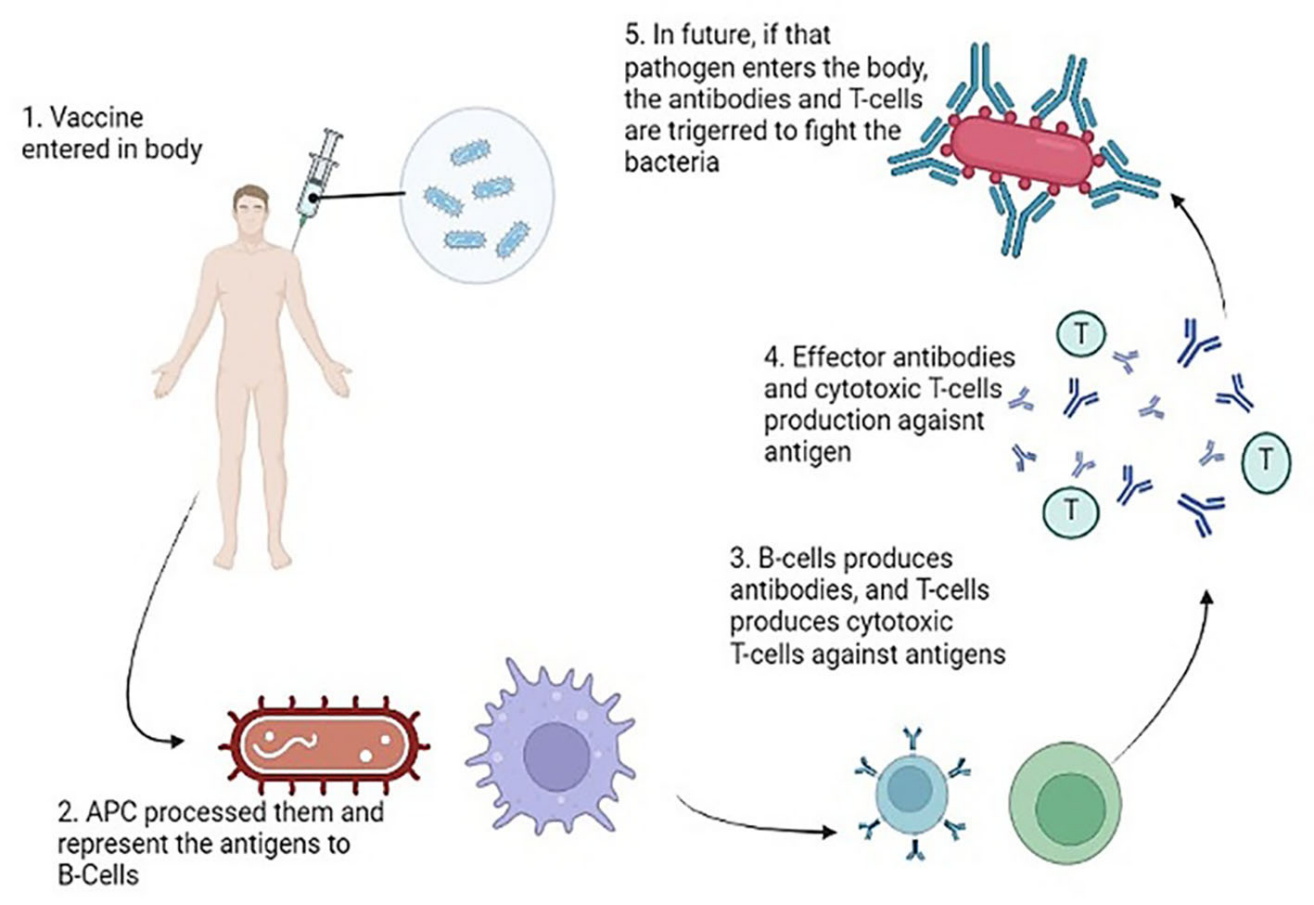
Mode of Action of Vaccines generating an immune response against the virulent components of bacteria.

Upon the entry of a pathogen into the body, antigen-presenting cells undertake the crucial task of processing and presenting these invaders to both B and T cells. The CD4+ T helper cells play a pivotal role by providing growth factors and signals to activate the B and T cells. Subsequently, these activated cells, recognizing the antigens, undergo proliferation, generating effector cells and memory cells. The effector cells, armed with the ability to recognize and target specific antigens, take action in various ways. B cells release antibodies against the identified pathogens, while Cytotoxic T cells execute the targeted destruction of the antigens (Mayer and Impens, 2021). The memory cells, on the other hand, store vital information for sustained immunity against the encountered pathogen and persist longer in diverse host tissues. These memory cells assume responsibility for mounting secondary responses upon reencountering specific pathogens. In the context of vaccines and antigens, the immune response typically engages both B and T cells. When a vaccine contains an antigen, plasma cells are stimulated to produce antibodies that specifically bind to toxins or pathogens. Simultaneously, CD8+ T cells come into play, restricting and diminishing the spread of infection. **Figure 8** is a pictorial representation of mode of actions of antibacterial vaccines (Bidmos et al., 2018).

### 3.5 Nanomaterials

In the realm of orthopedic surgery, the escalation of microbial resistance to current antibiotic therapies stands as a prominent contributor to implant failure and suboptimal clinical outcomes. However, recent strides in sophisticated antimicrobial nanotechnologies present promising opportunities to effectively combat resistant microbes and preclude the onset of resistance through specialized processes. Nanomaterials, with their programmable physicochemical properties, can be tailored to exhibit bactericidal, antifouling, and immunomodulating capabilities, enabling precise delivery of antibacterial agents to infection sites (Sharmin et al., 2021). Notably, nanoparticles (NPs) have emerged as innovative tools in the fight against severe bacterial diseases, circumventing conventional antimicrobial challenges, including antibiotic resistance (Imani et al., 2020).

#### 3.5.1 Mode of action of nanomaterials

Nanoparticles exhibit distinct physio-chemical characteristics that position them as formidable contenders in combating antibiotic resistance. Leveraging a variety of novel bactericidal pathways, nanomaterials demonstrate the capacity to exert antimicrobial activity through intricate mechanisms. They can adhere to bacterial membranes, inducing damage and facilitating the release of cytoplasmic components. Upon membrane penetration, nanomaterials can further target intracellular elements, including DNA, ribosomes, and enzymes, disrupting normal cellular functions. This interference results in electrolyte imbalance, oxidative stress, and enzyme inhibition, collectively leading to bacterial cell death (Sánchez-López et al., 2020). The core material, shape, size, and surface functionalization of nanomaterials dictate their bactericidal pathways. Early studies focused on modifying intrinsic core materials to create nanomaterials with diverse modes of action. For instance, antimicrobials based on silver nanoparticles utilize free Ag+ ions as the active agent (Wang et al., 2017).

**Figure 9:**
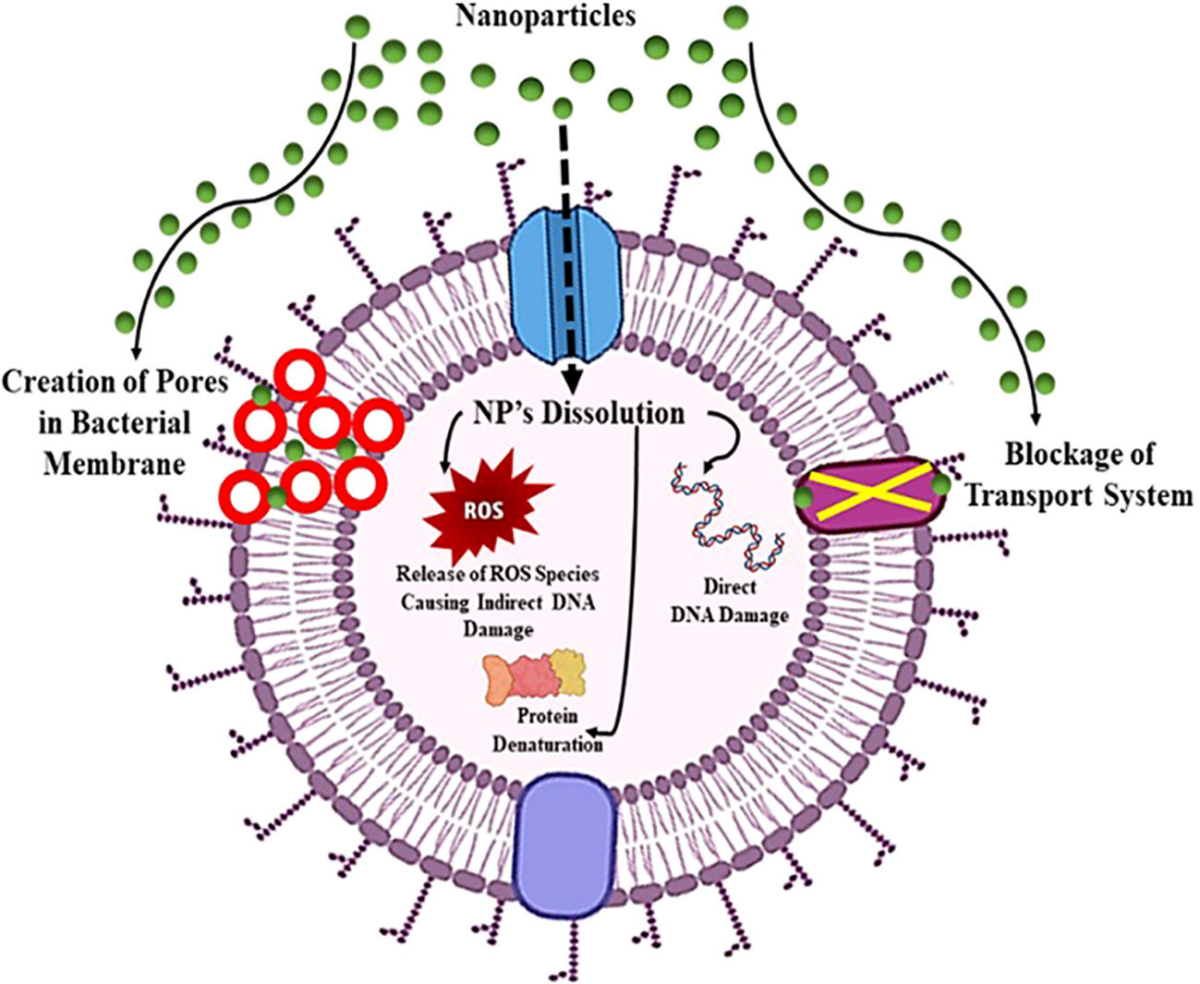
Mechanism of action of nanoparticles showing board spectrum antibacterial properties against pathogenic bacteria.

Silver ions damage DNA and impede electron transport across bacterial membranes. Copper nanoparticles generate reactive oxygen species (ROS), disrupting bacterial cells’ amino acid synthesis and DNA maintenance, akin to free Cu2+ ions. Meanwhile, ZnO and TiO2-based nanomaterials induce bacterial cell death by damaging cell membranes and producing ROS (Nisar et al., 2019). The versatility of nanomaterial cores offers a range of antibacterial actions against drug-resistant superbugs. However, unfunctionalized nanomaterials often exhibit limited antibacterial efficacy, restricting their biomedical applications due to low therapeutic indices (i.e., selectivity) against healthy mammalian cells. The surface chemistry of nanomaterials emerges as a pivotal factor in modulating their interactions with bacteria, enhancing broad-spectrum activity while concurrently reducing toxicity toward mammalian cells (**Figure 9**) (Jeevanandam et al., 2018). To elaborate on the mode of action against antibiotic-resistant bacteria, nanomaterials effectively disrupt key cellular processes in microorganisms, including interference with genetic material (DNA), inhibition of protein synthesis (ribosomes), and disruption of enzymatic activities. The multifaceted mechanisms employed by nanomaterials underscore their potential in addressing the challenge of antibiotic resistance.

### 3.6 Gene editing

*Pseudomonas aeruginosa* genome editing has been employed to address multidrug resistance through Specific I-F CRISPR-Cas, inducing reverse mutations in the PA154197, an epidemic multidrug-resistant genotype (Makarova et al., 2011). The most used Cas protein is Cas9, with specific gRNA followed by CRISPR to edit the genome (Hartenian and Doench, 2015).

This strategy aims to combat multidrug resistance by developing sensitivity to antibiotics, disrupting the outer membrane’s permeability. The edited *P. aeruginosa* strain exhibits remarkable susceptibility to antibiotics, offering a potential breakthrough in the battle against multidrug-resistant bacteria. In addition to genome editing, chimeric antigen receptor (CAR-T) cells have been engineered for Allogeneic Combination Immunotherapy to tackle multidrug resistance. Molecular seizer Transcription activator-like effector nucleases (TALEN) were employed to modify CAR-T cells for allogeneic adoptive transfer of multidrug-resistant T cells (Xu et al., 2019). This approach not only reduces alloreactivity but also enables these cells to withstand lymphodepleting regimens, enhancing their therapeutic potential.

Multidrug resistance mechanisms often involve transporter proteins, with ABC transporters playing a key role in mediating resistance (Locher, 2016; Adamska and Falasca, 2018). These transporters, spanning from ABCA to ABCG, facilitate the transport of chemotherapeutics in and out of bacterial cells. Notably, these ABC transporters are predominantly located in the cell membrane of bacteria, serving to transport essential nutrients. P-glycoprotein (P-gp), a transmembrane glycoprotein encoded by *ABCB1*, is implicated in multidrug resistance (Beis, 2015). In cancerous cells, the knockout of *ABCB1* enhances the sensitivity to multiple chemotherapeutic drugs, including alkaloids, taxanes, anthracyclines, and epipodophyllotoxins. Following the knockout, the increased sensitivity of *ABCB1* is demonstrated through the accumulation of doxorubicin and rhodamine 123 in tumor cells, presenting a potential strategy for overcoming multidrug resistance in cancer treatment (Yang et al., 2016).

#### 3.6.1 Mode of action of gene editing

**Figure 10:**
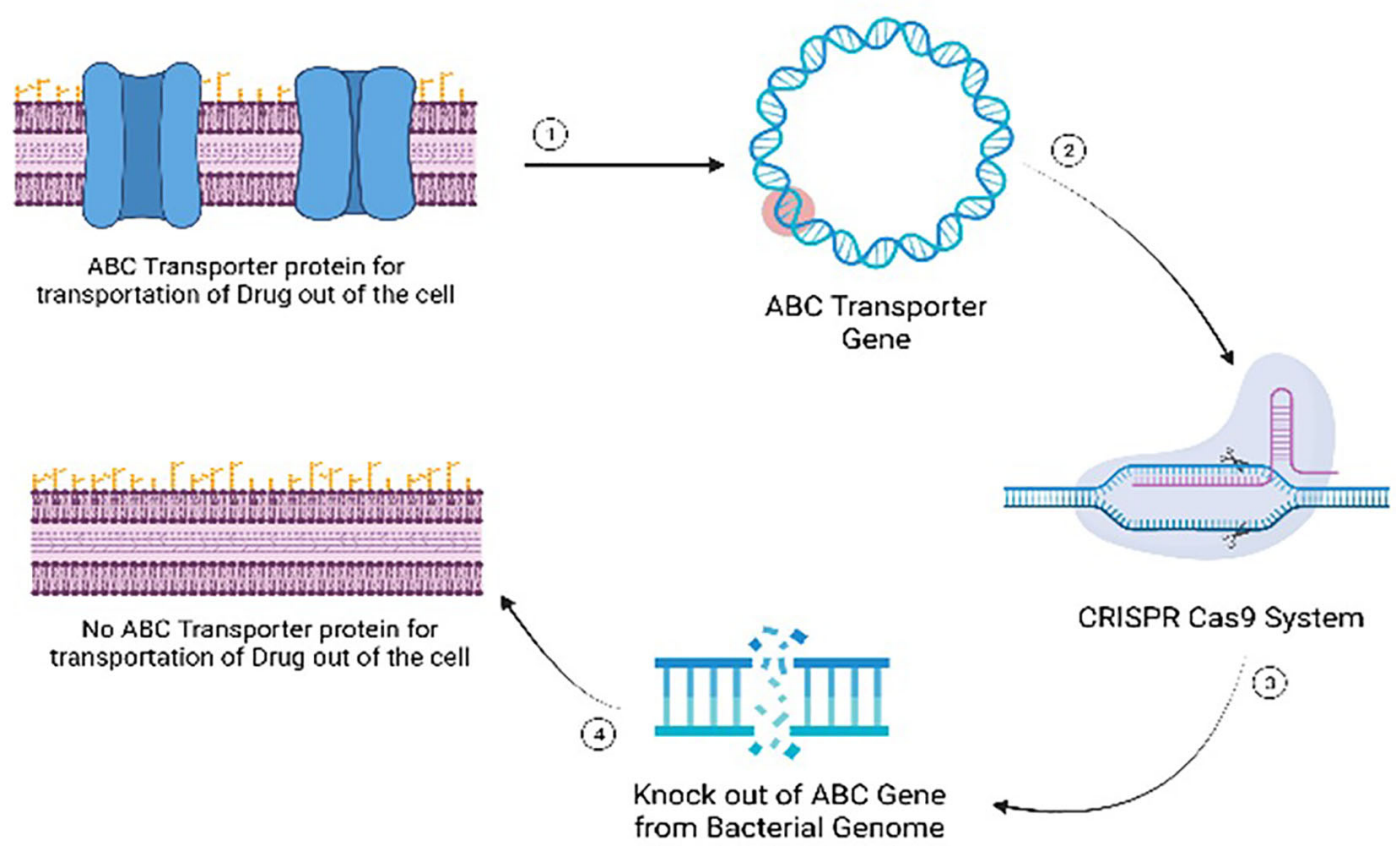
Mode of action of gene editing as an antimicrobial alternative for managing multi-drug resistant microbial infections.

Gene editing represents a revolutionary approach in addressing antibiotic-resistant bacteria by targeting specific genetic elements associated with resistance mechanisms. The primary mode of action involves the precise modification or disruption of genes responsible for conferring antibiotic resistance. For instance, the technique can be employed to target genes encoding antibiotic efflux pumps, which play a crucial role in expelling antibiotics from bacterial cells (Mohanty et al., 2021). By disrupting these pumps through gene editing, the bacteria’s ability to pump out antibiotics is compromised, rendering them more susceptible to the drugs. Another target includes genes involved in membrane permeability, a key factor in antibiotic resistance (Delcour, 2009). Gene editing can alter the structure or function of these genes, affecting the permeability of bacterial membranes and enhancing the penetration of antibiotics into the cells. Additionally, modifying genes related to drug targets can prevent bacteria from developing resistance by altering the target site’s structure (Luthra et al., 2018). This precise manipulation at the genetic level offers a nuanced and effective strategy to combat antibiotic resistance, providing hope for the development of novel antibacterial therapies. As advancements in gene editing technologies continue, the potential for tailored and targeted interventions against antibiotic-resistant bacteria becomes increasingly promising. (**Figure 10**).

## 4 Comparison of Methods in the curation of Bacterial infections

The effectiveness of fifth-generation antibiotics, bacteriocins, bacterial vaccines, Medicinal plants, nanomaterials, and gene editing in treating antibiotic-resistant bacteria varies based on their distinct mechanisms and applications. Fifth-generation antibiotics, characterized by improved spectrum coverage and reduced resistance development, have shown efficacy in addressing certain resistant strains. However, their impact is limited as bacteria can still evolve resistance over time (Fair and Tor, 2014). Bacteriocins, naturally occurring antimicrobial peptides produced by bacteria, exhibit promise in targeted bacterial inhibition. Their specificity allows for minimal harm to beneficial bacteria, but their application may be constrained by limited spectra of activity (Li et al., 2021). Medicinal plants offer a rich source of natural compounds with potential antimicrobial properties, providing a diverse array of bioactive molecules to combat antibiotic-resistant bacteria. However, their variable efficacy, dependence on specific plant species, and challenges in standardization present obstacles in harnessing their full potential as consistent and reliable treatments (Mickymaray, 2019). Bacterial vaccines, designed to stimulate the immune system against specific bacterial pathogens, offer a preventive approach to combat antibiotic resistance. While effective in preventing infections, their utility is restricted to specific bacterial strains, and the development of vaccines for all pathogens is challenging (Jansen et al., 2018). Nanomaterials, such as silver nanoparticles or nanotubes, present an innovative avenue by disrupting bacterial membranes and inhibiting growth. However, concerns about potential toxicity and environmental impact necessitate careful consideration (Ahmad et al., 2020). Gene editing techniques, on the other hand, provide a highly tailored and evolving strategy to address antibiotic resistance. By precisely targeting resistance genes, these techniques offer the potential to reverse or mitigate resistance mechanisms (Murugaiyan et al., 2022). The adaptability of gene editing allows for continuous refinement in response to emerging resistance patterns. Despite their promise, challenges include off-target effects and ethical considerations (Han et al., 2020). The comparison among the techniques mentioned above to combat multi-drug resistant bacteria is shown in **Table 1**.

**Table 1:** Comparison of techniques used to combat bacterial infections via *in-vitro* biological assays.

Bacteria	Technique	Efficacy		Efficiency		Validity/toxicity	Reference
Antibiotics							
Methicillin-resistant *Staphylococcus aureus*	Antibiotics extracted from Jordanian microbes (*Streptomyces* spp.) NF140 NF141 KTD123 (B*acillus* spp.) KTD119 KTD120 KTD133 NF131	Zone of inhibition(mm) 27 30 23 42 22 18 26					(Ishak et al., 2021)
Methicillin-resistant and suseptible *Staphylococcus aureus* *Streptococcus pyogenes* *Streptococcus agalactiae* *Escherichia coli* *Klebsiella pneumoniae* *Klebsiella oxytoca*	Ceftaroline fosamil (formerly PPI-0903, TAK-599)	MIC(g/ml) 2 2 2 2 2 2		99%		The half-life is around 2.5–3 hours	(Selvan and Ganapathy, 2016)
Cephalosporin-resistant *S. Pneumonia* Ampicillin-susceptible *E. Faecalis*	Ceftobiprole	MIC (µg/ml) 2 2		16%		The half-life is around 3–4 hours	(Selvan and Ganapathy, 2016)
Methicillin-resistant *Staphylococcus pseudintermedius*	Benzguinol A	MIC(μg/mL) 0.5-1					(Nocera et al., 2020)
Methicillin-resistant *Staphylococcus pseudintermedius*	Benzguinol B	0.5-1					(Nocera et al., 2020)
Methicillin-resistant *Staphylococcus pseudintermedius*	Amikacin	8-16					(Nocera et al., 2020)
Medicinal plants							
E. Coli ATCC8739 AG100 MC4100 W3100 *E. aerogenes* ATCC13048 EA27 EA294 *K. pneumoniae* ATCC11296 KP55 K2 *P. stuartuii* ATCC29916	*B. obscura*	MIC (µg/mL) 16 64 128 32 32 32 64 64 32 128 64		65.52%			(Touani et al., 2014)
*E. coli* ATCC8739 AG100A MC4100 W3100 *E. aerogenes* CM64 EA27 EA294 *K. pneumoniae* ATCC11296 KP55 K2 *P. stuartuii* ATCC29916	*P. fernandopoina*	256 512 1024 512 1024 1024 512 256 512 512 1024		72.41%			(Touani et al., 2014)
Methicillin-resistant *Staphylococcus aureus* MBL *A. baumannll*	*E. microphylla*	MIC (µg/mL) 1250 5000					(Cihalova et al., 2015)
*C. neoforman* *S* *P. aeruginosa* vancomycin-resistant *Enterococcus*	*Macaranga barteri*	75.625 165.244 114.026		50%			(Cihalova et al., 2015)
*P. aeruginosa* Methicillin-resistant *Staphylococcus aureus*	*Ciprofloxacin*	0.09 0.11		50%			(Cihalova et al., 2015)
Methicillin-resistant *Staphylococcus aureus* vancomycin-resistant *Enterococcus* *K. pneumonia* CRE P. aeruginosa MBL	*P. ophthalmicus*	275 137.5 137.5 137.5					(Magpantay et al., 2021)
*S. aureus* *B. geveus* *S. typhimurium* *E. coli*	*N. sattiva* (Essential oil)	Inhibition zone (mm) 15.7 14.0 26.5 30.3					(Bakal et al., 2017)
Gene editing							
Cocktail of *E. coli* phage ECP311, *K. pneumoniae* phage KPP235, and *Enterobacter* phage ELP140	CRISPR *Klebsiella pneumonia* *Enterobacter spp*	100% reduction after 5 doses; 90% survival					(Zimina et al., 2017)
*Enterococcus* PHIEF24C, PHIEF17H, and PHIM1EF22 phages	*Enterobacter spp*	Inhibition of growth					(Zimina et al., 2017)
Phage	*S*. *aureus*	4.076 ± 0.268					(Zimina et al., 2017)
Ointment consisting of the phage lysin lysgh15 and apigenin	*Staphylococcus aureus*	102CFU/mg at 18 h after treatment		a reduction of 3.3 log units			(Zimina et al., 2017)
ZNO NPS	MRSA Nanoparticles	16 and 17 mm at 500 μg/ml					(Zimina et al., 2017)
TIO2 NPS TIO2 NPS	MRSA Nanoparticles	14 mm at 500 μg/ml					(Zimina et al., 2017)
Bacteriocins							
MDRE and vancomycin-resistant *Enterococcus*	Bacteriocin *Enterococcus faecalis* (EF478)	The inhibition zone was 2-12 mm.		41.1%		Stability for 1 year at -20°C	(Phumisantiphong et al., 2017)
Methicillin-resistant *Staphylococcus aureus*	*S. epidermidis* (ATCC12228 and NCCP14768)	The inhibition zone was 10.6 ± 0.1mm.				Stability for 20 minutes at 45°C	(Jang et al., 2020)
MRE and vancomycin-resistant *Enterococcus*	*Enterococcus faecalis (*KT11)	The inhibition zone was 15-20 mm.				Stability for 3 months at -20 and -80°C	(Abanoz and Kunduhoglu, 2018)
ESBL *E. coli*	*B. subtilis* (SM01)	10.6 ± 1.16mm				Maximum antibacterial activity was at 37°C	(Mickymaray et al., 2018)
*B. mycoides* EMTC 9, *S. enterica* ATCC 14028, *M. luteus* EMTC 1860 *E. coli* ATCC 25922, *B. cereus* EMTC 1949, *A. faecalis* EMTC 1882 *P. vulgaris* ATCC 63, *P. fluorescens* EMTC 42, *C. albicans* EMTC 34.	*Lactobacillus plantarum (*B884*)* *Lactobacillus paracasei* (B2430) *Lactobacillus delbrueckii* (B2455)	10mm 11mm 11mm		14.1% 12.8% 4.6%		Incubation period: 18 hours 20 hours 54 hours	(Zimina et al., 2017)
*S. putrefaciens*	*Enterococcus faecalis* (EFL4)	MIC is 0.32 µg/mL				4°C for 8 days.	(Wright et al., 2016)
Gram-positive; *E. coli*	*L. plantarum (*BLp*)*	The inhibition zone is 30.2 mm				Stability for 15 mins at 50°C.	(Hassan et al., 2020)
*S. aureus* CICC10384 *E. coli CICC10302*	*L. rhamnosus XN2*	Inhibition zone 15.1 ± 1.2mm 7.2 ± 0.5mm				Stability for 2 months at 4°C	(Wei et al., 2022)
Methicillin-resistant *Staphylococcus aureus* Methicillin-vancomycin-resistant *S. warneri* vancomycin-resistant *Enterococcus*	*Enterococcus faecalis* KT11	Inhibition zone: 20 mm 20 mm 17 mm				Stability 90 days at -20°C and -80°C.	(Abanoz and Kunduhoglu, 2018)
*Pseudomonas stutzeri*	Bacteriocin H4 isolate from tiger prawn	Inhibition zone 887.10 ± 409.24 mm^2^/mL					(Feliatra et al., 2018)
*H. pylori* ZJC03	*Lactiplantibacillus plantarum* PLNC8	The inhibition zone was 7.06 ± 0.15mm at 80µM				Stability for 72 hours at 37°C	(Jiang et al., 2022)

## 5 Antibiotic stewardship advocacy

Antibiotic stewardship advocacy is crucial in addressing antibiotic resistance, emphasizing public education, responsible prescribing practices, and policy initiatives. Engaging healthcare providers through feedback mechanisms and policy initiatives, fostering international cooperation, and exploring innovative alternatives like phage therapy are integral to this multidimensional approach. Ongoing research and innovation remain essential for sustainable antibiotic use and global health (Majumder et al., 2020a; Majumder et al., 2020b). These initiatives encompass the regulation of antibiotic use in agriculture, healthcare settings, and the pharmaceutical industry. Collaborative efforts among healthcare institutions, research organizations, and pharmaceutical companies are highlighted for their role in developing and implementing responsible antibiotic use strategies, conducting research on antibiotic-resistant bacteria, and supporting the development of new antibiotics.

## 6 Conclusion

Multidrug-resistant organisms are bacteria that have developed resistance to specific treatments and can no longer be controlled or destroyed by them. Multidrug resistance in bacteria can be caused in one of two ways. These bacteria can accumulate many genes within a single cell, each of which codes for drug resistance to a single therapy. This type of accumulation is particularly common on-resistance (R) plasmids. Second, increased expression of genes encoding multidrug efflux pumps, which expel various drugs, can contribute to multidrug resistance. Antibiotic resistance is a big concern, as some bacteria have gained resistance to virtually all antibiotics now in use. These bacteria are a big public health concern because they have the potential to cause serious illness. Antibiotic-resistant gut bacteria include methicillin-resistant Staphylococcus aureus (MRSA), vancomycin-resistant Enterococcus (VRE), and multidrug-resistant Mycobacterium tuberculosis (MDR-TB), and carbapenem-resistant Enterobacteriaceae (CRE). Antimicrobial resistance is on the rise worldwide, and Antibiotic resistance has extended to various environmental niches and the introduction of superbugs has made it even more difficult to implement effective control measures. With advances in science and technology, this review exposes the most cutting-edge technologies, such as newly discovered medicinal plants, unique gene-editing techniques, novel nanomaterials, and bacterial vaccines, that will eventually aid in the fight against MDR bacteria in the future.

## Author contributions

MN: Supervision, Writing – original draft, Writing – review & editing. MW: Conceptualization, Methodology, Writing – original draft. IM: Conceptualization, Methodology, Supervision, Writing – original draft, Writing – review & editing. NA: Conceptualization, Methodology, Supervision, Writing – original draft, Writing – review & editing. FA: Writing – original draft. JH: Writing – original draft. HJ: Writing – original draft.
